# Retraction Note to: Effects of HDM2 antagonism on sunitinib resistance, p53 activation, SDF-1 induction, and tumor infiltration by CD11b+/Gr-1+ myeloid derived suppressor cells

**DOI:** 10.1186/s12943-021-01379-7

**Published:** 2021-06-11

**Authors:** David J. Panka, Qingjun Liu, Andrew K. Geissler, James W. Mier

**Affiliations:** 1 Division of Hematology-Oncology, Beth Israel Deaconess Medical Center and Harvard Medical School, Boston, MA USA; 2 Division of Urology, Beijing Friendship Hospital, Capital Medical University, Beijing, China; 3 330 Brookline Avenue, RW-571, Boston, MA 02215 USA; 4 330 Brookline Avenue, RW-563A, Boston, MA 02215 USA

**Retraction Note to: Mol Cancer 12, 17 (2013)**

**https://doi.org/10.1186/1476-4598-12-17**

The Editor-in-Chief has retracted this article after an investigation by Harvard Medical School and Beth Israel Deaconess Medical Center and additional analysis by the Office of Research Integrity found:

- Reuse and relabelling of the same source bands to falsely represent different experimental results in Figure 1 (reuse of the bands in the fourth to fifth lanes of the second row representing noxa expression in the control group of A498 cell type, to falsely represent noxa expression in the sunitinib resistant group of A498 cells in the same figure, eleventh to twelfth lanes of the second row)
- Reuse and relabelling of the same source bands to falsely represent different experimental results in Figure 1 (reuse of the band in the eighth lane of the second row representing noxa expression in the third sample of the sunitinib responding group, to falsely represent noxa expression in the fifth sample of the sunitinib responding group in the same figure, tenth lane of the second row
- Reuse and relabelling of the same source bands to falsely represent different experimental results in Figure 6B (first two lanes of the bottom row representing vinculin expression in control group, and eleventh and twelfth lanes of the bottom row representing vinculin expression in dox + sunitinib group)

Andrew K Geissler and James W Mier agree with this retraction, David J Panka does not agree with this retraction. The Editor-in-Chief has not been able to find a current email address for Qingjun Liu.

